# Towards inclusive medical education in Egypt: a cross-sectional study of sociocultural adaptation among international students

**DOI:** 10.1186/s12909-025-08232-1

**Published:** 2025-11-29

**Authors:** Tasneem Deibes, Mohammad Yaseen, Rodina S. Elkhouly, Sarah M. Yunis, Shady Aboheif, Ebrahim Hakam, Mohamed Aboskiena, Eman N. Metwally, Nouran Riad, Tariq M. Ibrahim, Mariam M. Awadh, Abdel-Hady El-Gilany, Heba Sheta, SCA team of collaborators, Abdulrahman Jamal, Nehal Ezzat, Shahd Ismail, Abdelaziz Mohamed, AhmedEltayeb Emad, Mohamed G. Elsayed, Moath Alhaj Ali, Khaled Abuzeid, Tengu Nurul Farihah Binti Tengku Nasrudin

**Affiliations:** 1 Faculty of Medicine, Mansoura University, Mansoura, Egypt; 2 Mansoura Students’ Scientific Association, Faculty of Medicine, Mansoura University, Mansoura, Egypt; 3 Medical Program, Mansoura University Hospitals, Mansoura, Egypt; 4 Public Health and Community Medicine Department, Faculty of Medicine, Mansoura University, Mansoura, Egypt; 5 Pathology Department, Faculty of Medicine, Mansoura University, Mansoura, Egypt

**Keywords:** Sociocultural adaptation, International medical students, Medical education, Egypt, SCAS-R, Cultural diversity

## Abstract

**Background:**

International medical students face unique challenges in adjusting to new sociocultural environments, particularly in resource-limited settings. In Egypt, despite calls for increasing cultural diversity in medical education, limited research has explored how international students adapt and what factors influence their experience. This study aimed to assess the sociocultural adaptation of international medical students at Mansoura University to inform targeted strategies enhancing their inclusion and well-being.

**Methods:**

A cross-sectional study was conducted among international medical students at Mansoura University between September and November 2024 using an online survey. Data were collected through convenience and snowball sampling, along with a non-probability proportionate quota based on academic year. The survey included sociodemographic items and the Sociocultural Adaptation Scale–Revised (SCAS-R), a validated tool that assesses five aspects of sociocultural adaptation on a five-point Likert scale.

**Results:**

A total of 395 international students participated, with a mean SCAS-R adaptation score of 3.37 (SD = 0.69). Adaptation was highest among 1st-year students (3.52) and lower in subsequent years (2nd: 3.21; 3rd: 3.39; 4th: 3.31; 5th: 3.48). Males reported higher scores in personal interest/community involvement (3.09 vs. 2.86, *p* = 0.011) and language proficiency (4.25 vs. 4.00, *p* = 0.036). Arab students had significantly higher overall adaptation than non-Arabs (3.40 vs. 3.21, *p* = 0.046), primarily due to stronger language proficiency (4.42 vs. 2.76, *p* < 0.001). Students with sufficient income showed better adaptation in communication (3.41 vs. 3.15, *p* = 0.021), ecological adjustment (3.32 vs. 2.99, *p* = 0.008), and language (4.18 vs. 3.83, *p* = 0.039).

**Conclusion:**

The findings of this study emphasize the need for medical institutions to implement inclusive support strategies, including bilingual language training, targeted financial assistance, and sustained integration efforts beyond the first academic year.

**Trial registration:**

Not applicable.

**Supplementary Information:**

The online version contains supplementary material available at 10.1186/s12909-025-08232-1.

## Introduction

According to Berry et al., sociocultural adaptation is the dynamic process of how people manage and adjust to their lives in new cultural settings [1]. It includes the capability to cope and utilize a range of multicultural knowledge, skills, and attributes during intercultural communication in order to facilitate integration into the host culture [2]. International students, therefore, represent a population for whom sociocultural adaptation is particularly critical, as they must navigate not only academic demands but also the challenges of language, cultural norms, and social integration in an unfamiliar environment. This is particularly true for students pursuing international medical education, where rigorous academic demands and significant psychological stressors intensify the challenges of adapting to a new cultural setting [3, 4].

Indeed, research on the sociocultural adaptation of international medical students in Europe [5], North America [6], and Asia [7] consistently highlights common challenges experienced by the students such as language barriers, perceived discrimination, homesickness, and separation from family. In particular, lower levels of host-country language competence have been associated with higher acculturative stress [8, 9], as language and cultural differences hinder students’ ability to navigate academic communication, especially in group assignments [10]. Furthermore, given the essential role of language for expanding social networks, international students' psychological and physical well-being may be at risk when their level of language proficiency impedes their help-seeking methods [11]. However, most of this evidence comes from high-income settings, while studies in Low- and Middle-Income Countries (LMICs) remain scarce. This gap is especially relevant to Egypt, where medical students already face considerable mental health challenges, including psychological distress, exhaustion, and burnout, compounded by intense academic demands that limit recreational time and disrupt work–life balance [12, 13].

Recognizing the significant difficulties international medical students face during their education, medical educators have repeatedly called for proper inclusivity and cultural diversity in medical education. After all, cultural diversity is a cornerstone aspect of graduating culturally competent healthcare providers [14]. It brings valuable cultural assets such as varied life experiences and perspectives that enhance students’ ability to deliver culturally sensitive care and contribute to reducing health disparities [15]. Moreover, exposure to diverse peers fosters the development of unconscious cultural competence through interaction and dialogue, enriching students’ understanding of different cultural backgrounds [16]. The need for inclusive educational environments is particularly true for countries such as Egypt, which has become an appealing destination for students from both neighboring and distant countries reaching a total of 51649 inbound international students in 2024 [17]. This is partly due to the relatively low cost of living and education, the presence of internationally accredited medical schools, and its shared cultural and religious background [18]. Egypt also hosts students from non-Arab countries, who come with an additional set of challenges, including language and cultural barriers [18].

Unfortunately, despite international calls for inclusivity and cultural diversity in medical education, and the increasing numbers of international medical students in Egyptian Universities, the structural support systems that help them in sociocultural adaptation often remain lacking to meet students' needs. While some initiatives exist, such as Mansoura University’s orientation days for new incoming international medical students, these are typically limited to initial welcome activities without sustained follow-up or long-term integration support. Studies at other Egyptian institutions, like Tanta University, reveal that many international students face significant difficulties in sociocultural adaptation, particularly in terms of cultural isolation, language barriers, and lack of ongoing mentorship [19]. Similarly, another research on acculturative stress in an Egyptian medical school highlights persistent cultural challenges, including perceived discrimination and academic pressures, particularly among non-Arab and female students [20].

Given the gap between the growing international student population and the limited institutional support, particularly in Arabic-speaking LMICs where evidence remains scarce, this study aims to assess Mansoura University’s international medical students’ sociocultural adaptation and its relation to their sociodemographic backgrounds. By addressing this gap, the study provides novel insights from an underrepresented context and generates evidence to inform targeted strategies that foster integration, inclusivity, and well-being.

## Methods

### Study design and study period

A descriptive cross-sectional study with an analytical component was conducted among international medical students. The study was conducted in the Faculty of Medicine, Mansoura University, Egypt, between September and November 2024. This study followed the STROBE guidelines for reporting observational studies [21].

### Study population

The study population included international medical students enrolled at the Faculty of Medicine, Mansoura University, during the 2024–2025 academic year. This encompassed students from both the Traditional program and the Mansoura Manchester Program for Medical Education, across the first to fifth academic years. According to official records from the Students’ Affairs Department, approximately 3572 international students were enrolled during this period.

A non-probability quota sampling technique was employed to ensure proportional representation across academic years. Quotas were established based on the actual distribution of international students in each year.

### Pilot study and sample size

A pilot study involving 30 international students was conducted to assess clarity and determine the standard deviation of sociocultural adaptation scores to calculate the sample size. Based on the pilot study’s participants' feedback, a few words in the English version of the Revised Sociocultural Adaptation Scale (SCAS-R) [22], were reported to be difficult, and their Arabic translation was included between parentheses in the final version of the survey. Since the survey was changed based on the feedback of the pilot study, these responses were not included in the final analysis.

The sample size was calculated using MedCalc 23.2.1 software [23], considering the mean score on the SCAS-R scale as the primary outcome of interest. Our pilot study reported a mean sociocultural adaptation score of 3.29 with a standard deviation of 0.819. With a margin of error of 5% and a study power of 80%, the calculated required sample was 208. To account for convenience sampling, we used a design effect of 1.5, yielding a total sample size of 312. In total, 408 responses were collected. Thirteen responses were excluded; ten participants did not consent to share their data, and three were submitted by Egyptian respondents rather than international students, leaving a total of 395 responses.

### Sampling and data collection approach

After determining the required sample size, the study used a combination of convenience and snowball sampling, along with a non-probability proportionate quota technique based on academic year. Data was collected using a self-administered questionnaire using Google Forms. The survey was shared with all international students through official groups via the Telegram app and other social media platforms. A team of collaborators was assembled and trained by the authors to assist in data collection. Participation was anonymous, voluntary, and completed at the participants’ convenience without compensation. To minimize missing data, all questions in the Google Form were mandatory, so students could not submit the survey without completing all items. Consequently, there were no missing data in the final dataset.

### Study tools

A structured self-reported online questionnaire was distributed to the faculty’s international students, which consisted of two sections.

The first included the SCAS-R [22], with the minor aforementioned adaptations based on the pilot study, which was used to assess the level of adaptability of international medical students to their current environment. The tool comprises 21 questions and was set to measure interpersonal communication, academic performance, personal interests & community involvement, ecological adaptation, and language proficiency. These items were scored on a five-point Likert scale ranging from 1 (not at all competent) to 5 (extremely competent). A total score was calculated by summing the responses across all items (possible range: 21 to 105), then dividing by the number of items to yield a final score ranging from 1 to 5. Higher scores reflect greater levels of sociocultural adaptation. The SCAS-R is a valid tool for measuring sociocultural adaptation and has a good test–retest reliability (*r* = 0.92) [22]. Additionally, the pilot study resulted in a Cronbach’s α of 0.93, indicating high internal consistency of the scale in line with that of the original scale (α = 0.92) [22, 24].

The second section of the questionnaire collected non-identifying sociodemographic characteristics, which included: age, sex, academic year, nationality, and self-perceived income sufficiency, assessed by the question: “Your source of income: less than sufficient/sufficient/more than sufficient.”

### Statistical analysis

Statistical analysis was conducted using Jamovi 2.3.28 software, and the figure was made using R programming software. Sociodemographic data were summarized using descriptive statistics. Categorical variables were reported as frequencies and percentages, while continuous variables were reported as mean and standard deviation (SD). Normality was assessed using a Quantile–Quantile (Q-Q) plot. An Independent Samples T-test and One-Way Analysis of Variance (ANOVA) were employed to compare the means of independent groups and determine whether significant differences existed among them. For variables found to be significant in the ANOVA, post hoc analysis was conducted using the Games-Howell test. The relationship between sociocultural adaptation scores and participants’ sociodemographic characteristics was examined using Pearson’s correlation. Additionally, factors associated with sociocultural adaptation scores were identified through linear regression analysis. A p-value less than 0.05 was considered statistically significant.

## Results

### Sociodemographic characteristics of the study participants

A total of 395 international students participated in this survey. Participants were nearly equally distributed across gender and academic years, with 2nd year students representing the highest proportion of the participants (24.8%), and 3rd year students were the lowest (15.4%). Most of the study participants were from Arabic countries (*n* = 327, 82.8%), mostly from Syria (23.8%), Sudan (17.7%), and Palestine (11.1%), while 17.2% of the participants were non-Arabs, mostly from Malaysia (*n* = 59, 14.9%). Most (86.6%) of the students perceived their income as sufficient, possibly reflecting the comparatively lower expenses of living and studying in Egypt (Table 1).

**Table 1:** Participants’ sociodemographic characteristics (*N* = 395)

Characteristics			n (%)
**Overall**			395 (100)
**Age**	21 or less		250 (63.3)
	Older than 21		145 (36.7)
**Sex**	Male		213 (53.9)
	Female		182 (46.1)
**Academic year**	1st year		64 (16.2)
	2nd year		98 (24.8)
	3rd year		61 (15.4)
	4th year		89 (22.5)
	5th year		83 (21.0)
**Nationality**	Arab country	Syria	94 (23.8)
		Sudan	70 (17.7)
		Palestine	44 (11.1)
		Bahrain	37 (9.4)
		Jordan	28 (7.1)
		Saudi Arabia	19 (4.8)
		Yemen	15 (3.8)
		Algeria	7 (1.8)
		Others*	13 (3.3)
		Total Arabs	327 (82.8)
	Non-Arab country	Malaysia	59 (14.9)
		Somalia	3 (0.8)
		Nigeria	3 (0.8)
		Philippines	1 (0.3)
		Indonesia	1 (0.3)
		Canada	1 (0.3)
		Total Non-Arabs	68 (17.2)
**Income sufficiency**	Sufficient		342 (86.6)
	Less than Sufficient		53 (13.4)

^*^Others: Iraq, Kuwait, Lebanon, Libya, South Sudan, and the United Arab Emirates (each had a frequency of 3 or less)

### Association between sociodemographic characteristics and SCAS-R scores

**Fig. 1:**
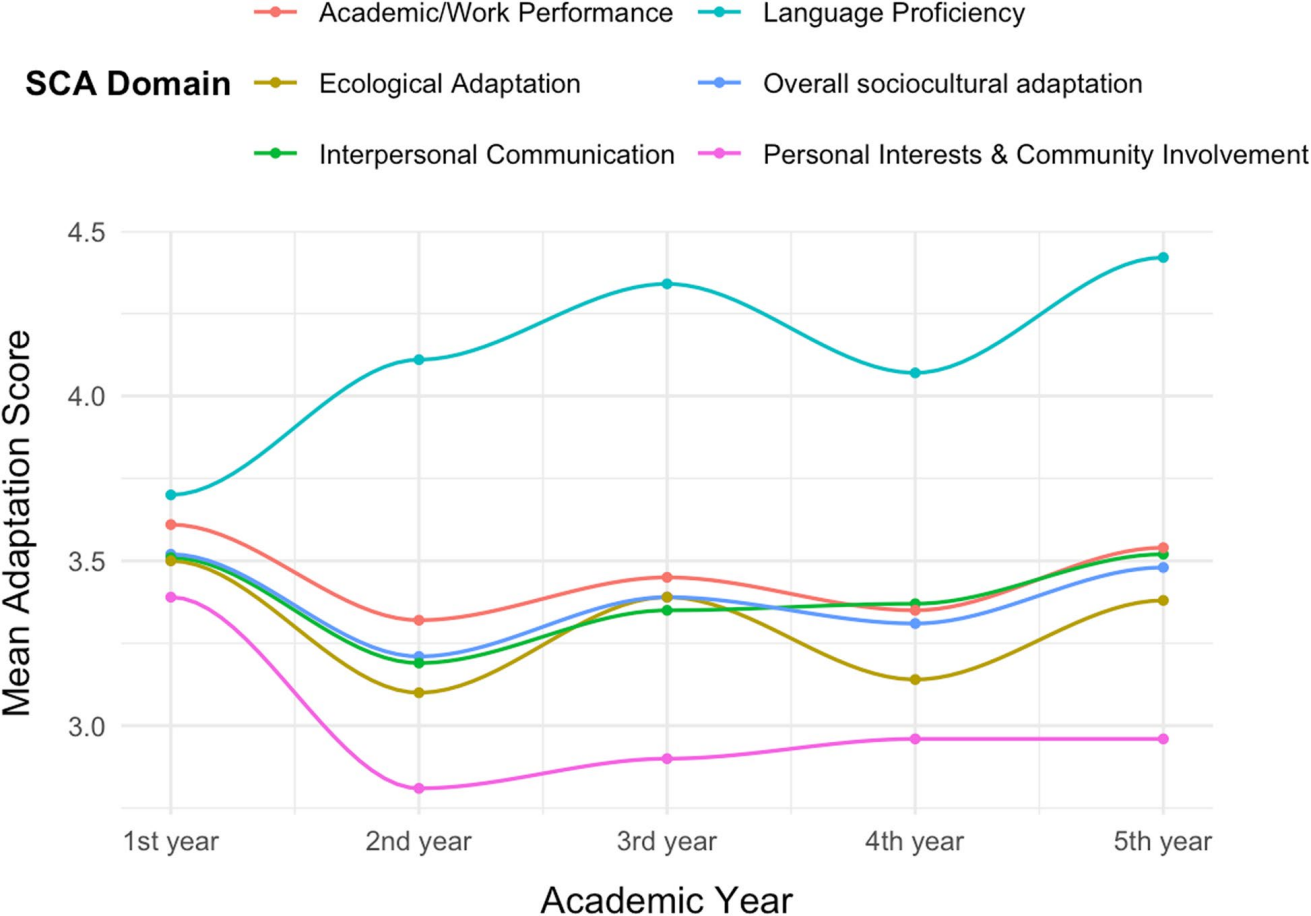
Variation in sociocultural adaptation domains across academic years

The overall mean SCAS-R score was 3.37 (SD = 0.69) (Table 2). Males displayed significantly higher adaptation scores particularly in personal interest & community involvement (3.09 vs 2.86, *p* = 0.011) and language proficiency (4.25 vs 4.00, *p* = 0.036) than females. First-year students reported the highest mean overall SCAS-R score (M = 3.52, SD = 0.72), which declined significantly among second-year students (M = 3.21, SD = 0.62). However, the subscales exhibited unique trajectories across academic years. Language Proficiency was the highest-scoring domain and showed a generally positive trend, increasing from a mean of 3.70 in the first year to a peak of 4.42 in the fifth year. In contrast, Personal Interests & Community Involvement was the lowest-scoring domain, dropping sharply from the first year (M = 3.39) to its lowest point in the second year (M = 2.81) before showing a slight and gradual recovery. The remaining domains followed a pattern similar to the overall score, characterized by a notable dip in the second year followed by a gradual recovery in subsequent years. All trends are visualized in Fig. 1.

**Table 2:** Comparison of SCAS-R scores across sociodemographic characteristics among international medical students at Mansoura University (*N* = 395)

Characteristics		Overall sociocultural adaptation^1^	Interpersonal Communication^1^	Academic/Work Performance^1^	Personal Interests & Community Involvement^1^	Ecological Adaptation^1^	Language Proficiency^1^
**Overall**		3.37 (0.69)	3.38 (0.77)	3.44 (0.86)	2.98 (0.88)	3.28 (0.86)	4.13 (1.15)
**Age**	≤ 21	3.36 (0.65)	3.37 (0.73)	3.43 (0.83)	3.00 (0.85)	3.27 (0.84)	4.13 (1.14)
	> 21	3.37 (0.76)	3.40 (0.84)	3.44 (0.91)	2.95 (0.92)	3.29 (0.89)	4.14 (1.17)
	*p*-value	0.931	0.706	0.934	0.584	0.851	0.925
**Sex**	Male	3.42 (0.72)	3.40 (0.80)	3.49 (0.84)	3.09 (0.90)	3.30 (0.89)	4.25 (1.10)
	Female	3.31 (0.66)	3.35 (0.74)	3.37 (0.87)	2.86 (0.84)	3.25 (0.83)	4.00 (1.19)
	*p*-value	0.111	0.599	0.159	**0.011**	0.602	**0.036**
**Academic year**	1 st year	3.52 (0.72)	3.51 (0.78)	3.61 (0.77)	3.39 (0.92)	3.50 (0.86)	3.70 (1.21)
	2nd year	3.21 (0.62)^**(a)**^	3.19 (0.72)^**(b)**^	3.32 (0.91)	2.81 (0.74)^**(a)**^	3.10 (0.75)^**(a)**^	4.11 (1.15)
	3rd year	3.39 (0.50)	3.35 (0.64)	3.45 (0.67)	2.90 (0.69)^**(a)**^	3.39 (0.69)	4.34 (0.97)^**(c)**^
	4th year	3.31 (0.81)	3.37 (0.85)	3.35 (0.93)	2.96 (0.96)^**(a)**^	3.14 (1.01)	4.07 (1.22)
	5th year	3.48 (0.71)	3.52 (0.78)	3.54 (0.88)	2.96 (0.96)	3.38 (0.89)	4.42 (1.06)^**(c)**^
	*p*-value	**0.026**	**0.029**	0.166	**0.002**	**0.009**	**0.003**
**Nationality**	Arab country	3.40 (0.72)	3.40 (0.81)	3.45 (0.91)	2.97 (0.91)	3.26 (0.89)	4.42 (0.97)
	Non-Arab country	3.21 (0.49)	3.29 (0.57)	3.38 (0.56)	3.03 (0.72)	3.34 (0.70)	2.76 (0.97)
	*p*-value	**0.046**	0.284	0.515	0.664	0.497	**< 0.001**
**Income sufficiency**	Less than Sufficient	3.40 (0.68)	3.15 (0.82)	3.28 (0.95)	2.80 (0.87)	2.99 (0.90)	3.83 (1.24)
	Sufficient	3.14 (0.73)	3.41 (0.76)	3.46 (0.84)	3.01 (0.88)	3.32 (0.85)	4.18 (1.13)
	*p*-value	**0.011**	**0.021**	0.161	0.097	**0.008**	**0.039**

^1^Mean (SD)

^**(a)**^Significantly less than 1 st years, *p*-value < 0.05

^**(b)**^Significantly less than 5th years, *p*-value < 0.05

^**(c)**^Significantly higher than 1 st years, *p*-value < 0.05

Arab students had significantly higher mean SCAS-R scores than non-Arab students (3.40 vs 3.21, *p*-value = 0.046), with this difference being largely attributed to differences in language proficiency (4.42 vs 2.76, *p* < 0.001). In addition, students with sufficient income showed less difficulty in sociocultural adaptation in terms of interpersonal communication (3.41 vs 3.15, *p* = 0.021), ecological adaptation (3.32 vs 2.99, *p* = 0.008), and language proficiency (4.18 vs 3.83, *p* = 0.039) than students with insufficient income (Table 2).

### Multivariate associations between sociodemographic characteristics and SCAS-R scores

In the multivariate linear regression analysis (Table 3), income sufficiency was the only significant predictor of overall sociocultural adaptation (β = 0.14, 95% CI [0.04, 0.24], *p* < 0.01). Nationality showed the strongest association with language proficiency, with Arab students reporting significantly higher proficiency (β = 0.55, 95% CI [0.47, 0.64], *p* < 0.001). Academic year was positively associated with language proficiency (β = 0.13, 95% CI [0.03, 0.24], *p* < 0.05) but negatively associated with personal interests and community involvement (β = − 0.14, 95% CI [−0.27, −0.02], *p* < 0.05). Female gender was also negatively associated with personal interests and community involvement (β = − 0.14, 95% CI [−0.25, −0.04], *p* < 0.01).

**Table 3:** Multivariate linear regression of factors associated with SCAS-R scores among international medical students at Mansoura University

Variable	Overall sociocultural adaptation^a^	Interpersonal Communication^a^	Academic/Work Performance^a^	Personal Interests & Community Involvement^a^	Ecological Adaptation^a^	Language Proficiency^a^
**Age (continuous)**	0.07 (−0.06, 0.19)	0.07 (−0.06, 0.20)	0.09 (−0.04, 0.21)	0.09 (−0.03, 0.22)	0.04 (−0.08, 0.17)	−0.08 (−0.18, 0.02)
**Sex**	−0.07 (−0.17, 0.04)	−0.02 (−0.13, 0.08)	−0.07 (−0.17, 0.03)	−0.14 (−0.25, −0.04)**	−0.04 (−0.15, 0.06)	0.03 (−0.06, 0.11)
**Academic year**	−0.04 (−0.17, 0.09)	−0.00 (−0.13, 0.12)	−0.06 (−0.19, 0.06)	−0.14 (−0.27, −0.02)*	−0.05 (−0.17, 0.08)	0.13 (0.03, 0.24)*
**Nationality**	0.09 (−0.01, 0.20)	0.05 (−0.05, 0.16)	0.02 (−0.08, 0.12)	−0.04 (−0.15, 0.06)	−0.03 (−0.14, 0.07)	0.55 (0.47, 0.64)***
**Income sufficiency**	0.14 (0.04, 0.24)**	0.12 (0.02, 0.22)*	0.08 (−0.02, 0.18)	0.10 (0.00, 0.20)*	0.14 (0.04, 0.24)**	0.14 (0.06, 0.22)**

Categorical variables were included in the model as dummy variables: Nationality (0 = Non-Arab, 1 = Arab), Sex (0 = Male, 1 = Female), Academic year (1–5), and Income sufficiency (0 = Less than sufficient, 1 = Sufficient)

^*^p-value < 0.05

^**^p-value < 0.01

^***^p-value < 0.001. CI: Confidence Intervals

^a^ β (95% CI)

## Discussion

Adjusting to life in a new cultural setting comes with a substantial set of challenges, which are further exacerbated by the demanding nature of medical education [25]. Evaluating international medical students’ adaptation to new settings and understanding the factors that shape it is key in supporting their academic and personal success [5]. It is also imperative to ensure an inclusive learning environment that caters to the culturally diverse student population in Egypt [26, 27]. Hence, this study aimed to assess Mansoura University’s international medical students’ sociocultural adaptation and how it relates to their socio-demographic backgrounds, providing recommendations for a more welcoming academic setting.

Using the SCAS-R, the mean total sociocultural adaptation score of our study’s participants was 3.37. This was slightly less than the scores reported among international students in Hong Kong (3.53) [7], Turkey (3.57) [28] and at a metropolitan university in the USA (3.50) [6]. The comparatively lower adaptation observed in our sample may be due to differences in sample characteristics, as the aforementioned studies assessed international students in general rather than medical students specifically. This is further supported by the findings of Shehata et al. [19], who reported a mean sociocultural adaptation score of 3.3 among Malaysian medical students in Tanta University in Egypt.

The lower sociocultural adaptation scores among medical students may be attributed to the intense academic demands of medical education, which significantly hinder students’ engagement with their communities, thereby affecting their adaptation to new cultures [5, 29]. This is further evidenced by the personal interests & community involvement scores among our study’s participants, which were the lowest across the SCAS-R subscales. This finding is of serious consequence, given that community involvement and interpersonal communication both play a critical role in helping students adapt to cultures [30].

Specifically, engaging in the community through participation in extracurricular activities and volunteering has shown to foster a strong sense of belonging among medical students and to enhance soft skills such as teamwork and communication [31]. Consequently, this involvement promotes the formation of meaningful interpersonal relationships with students of the host culture, providing international students with much-needed social support systems in the absence of their families and social networks [30]. These relationships in turn become crucial for navigating cultural transitions, and ultimately facilitating successful sociocultural adaptation [32]. Notably, the significantly higher sociocultural adaptation scores among male students were largely a reflection of higher personal interests & community involvement scores compared to females. This is also consistent with similar studies conducted in Egypt which highlight lower sociocultural adaptation [19], higher acculturative stress [20] and increased social media [33] use among female students compared to their male counterparts. This may be due to males having more opportunities to engage in Egyptian culture as compared to females who may face more constraints in doing so. Similarly, the lower adaptation scores observed among fourth-year students may reflect their reduced opportunities for community engagement during the early years of medical school as a result of pandemic restrictions [34]. Collectively, these findings underpin the importance of encouraging early community engagement as a key strategy to support international students’ adaptation and integration into the host culture.

In our study, a pattern resembling the U-curve model of cultural adjustment described by Lysgaard et al. was observed when comparing academic cohorts [35]. Mean scores for personal interests and community involvement were highest among first-year students, significantly lower for second-year students, and comparatively higher for students in their third, fourth, and fifth years. This trend was also noted in interpersonal relationships and overall sociocultural adaptation scores. The elevated first-year scores likely reflect the “honeymoon phase”; the initial stage of arriving to a new cultural environment. During this phase, sojourners are captivated by the host culture, open to new experiences and have great motivation to integrate into the host culture [36]. In second year, students appear to enter the “shock phase,” as the novelty fades and academic pressures and social fatigue set in; thereafter, a “recovery phase” sees adaptation scores rise again [20, 37]. Nevertheless, this interpretation must be considered with caution, as the cross-sectional design limits our ability to draw conclusions about longitudinal changes within individual students. Furthermore, Chien et al. contends that the U-curve oversimplifies the acculturation process, which is influenced by a complex interplay of individual, cultural, and contextual factors [38]. Future longitudinal research is needed to confirm these temporal trends.

Another interpretation for the higher adaptation scores among first years is that universities often focus integration efforts -such as mentoring schemes and structured social events- primarily on first-year students [39, 40]. While such programs prove to be effective for first year students’ adjustment, sustained engagement is recommended in further years to maintain the adaptation gains [41]. Accordingly, targeted support and campus services should be extended into the intermediate academic years to prevent the marked decline in sociocultural adaptation that can undermine both student well-being and academic performance.

The overall sociocultural adaptation score was also significantly influenced by nationality, with non-Arab students reporting lower adaptation levels compared to their Arab counterparts. This difference may be attributed to the language and cultural barriers faced by non-Arab students in a predominantly Arab environment like Egypt, which may hinder their participation in social interactions and integration within the local community. This interpretation is further supported by subscale findings, as non-Arab students scored significantly lower in both language proficiency and interpersonal communication compared to Arab students. Previous studies among international medical students in Egypt further support this notion. In a study by Shethata et al. in Tanta university [19], Malaysian students proficient in Arabic reported significantly higher adaptation scores. Meanwhile, Ali et al. in Ain Shams University identified language deficiencies and nationality as key sources of acculturative stress [20], highlighting the central role of language in the adaptation process.

Language difficulties are well documented as a significant barrier to sociocultural adaptation in various international contexts, including Australia [29], Hungary [32], USA [6], Malaysia [42] and Saudi Arabia [43]. Such challenges are particularly concerning in medical education, where language proficiency is critical for both academic success and clinical communication [5, 25, 44, 45]. Non-Arab medical students, in particular, have repeatedly reported language barriers as a major challenge, especially in clinical settings where communication with Arabic-speaking patients is essential [18, 45]. In fact, the significantly higher adaptation scores among participants with sufficient income may have been largely due to their ability to afford language courses, which corresponded with notably higher language proficiency scores.

Given the critical role of language in not only the sociocultural adaptation of international medical students but also their overall academic and clinical success, medical institutions should prioritize native language training as a core component of their integration efforts [46]. One effective approach is the adoption of bilingual language programs. In Egypt, medical education is primarily delivered in English—the global language of medicine—while clinical interactions typically occur in Arabic. Non-Arab students may therefore benefit from a bilingual curriculum that maintains English as the medium of instruction while incorporating targeted Arabic training to ease the transition into clinical environments [44].

In addition to bilingual strategies, foreign language support programs have shown promising results in other health education contexts. For instance, Seibold et al. reported significant improvements in clinical communication skills among nurses whose first language was not English after they received tailored English language instruction [47]. While several similar programs have demonstrated positive outcomes, the development of context-specific, needs-based language interventions remains essential [48]. Another proposed program directed at medical students is the English for Medical Purposes (EMP) program for international medical graduates in Canada, which aims to enhance communication skills in medical contexts [49].While such initiatives may face logistical and financial constraints, they have been shown to strengthen international students’ academic confidence and facilitate relationship-building with host-country peers and patients which is an essential factor linked to greater self-reported satisfaction, enhanced social connectedness, and ultimately, improved sociocultural adaptation [50].

Income sufficiency also emerged as a significant predictor of overall sociocultural adaptation, aligning with existing literature that links financial stress to reduced well-being among international students [51] and as a key contributor to acculturative stress [9, 20]. In our study, students reporting a less than sufficient income also scored lower on the ecological adaptation subscale, suggesting that students with adequate financial resources are better able to navigate the day-to-day practical demands of life in Egypt. This may reflect their greater ability to secure comfortable housing, access local services, and engage with the host environment without the added burden of financial stress all of which are central to ecological adjustment.

In recent years, Egypt has experienced significant inflation, with rates reaching 28.3% in 2024, contributing to rising rent, tuition fees, and daily living costs for both local and international students [52]. These economic pressures may disproportionately impact international students, many of whom rely on private rentals for accommodation [20]. Accordingly, medical schools and higher education institutions should prioritize the expansion of financial support mechanisms. These may include increasing scholarship opportunities for international students, implementing flexible tuition payment plans, and providing part-time work opportunities on campus. Such interventions can alleviate financial stress, promote student well-being, and support greater sociocultural engagement.

### Strengths and limitations

Our study, conducted in a resource-constrained setting amid rising inflation and economic instability, provides practical relevance and applicable findings, particularly for similarly affected regions.

However, this study is not without limitations. Conducting the study in a single faculty limits the generalizability of the findings to the broader population of international medical students in Egypt. Future research should include multiple institutions and non-medical students across the country to better capture nationwide trends. Moreover, its cross-sectional design limits the ability to infer causality; therefore, longitudinal research is recommended to better understand changes in adaptation over time. Additionally, the use of convenience and snowball sampling may have introduced selection bias, potentially leading to an overrepresentation of students who are more socially connected or motivated to participate, and underrepresentation of those who are more isolated or disengaged. This could have resulted in a sample that is not fully representative of the broader international student population. Future studies should consider employing randomized sampling strategies. The reliance on self-reported data also poses a risk of recall or social desirability bias, which may have influenced how participants rated their adaptation experiences.

## Conclusions

The present study provides important insights into the sociocultural adaptation of international medical students in a single Egyptian faculty. Key sociodemographic factors—including gender, nationality, income sufficiency, and academic year—were found to significantly influence adaptation levels. These findings highlight the need for targeted institutional support, particularly in the areas of language training, community engagement, and financial assistance, to foster inclusive and culturally responsive learning environments that promote student well-being and success.

## Supplementary Information

Supplementary Material 1.

## Data Availability

The data of this study is available from the corresponding author upon reasonable request.

## Abbreviations

ANOVA: Analysis of Variance
CI: Confidence Interval
EMP: English for Medical Purposes
LMIC: Low- and Middle-Income Country
Q-Q: Quantile–Quantile
SCAS-R: Sociocultural Adaptation Scale–Revised
SD: Standard Deviation
STROBE: Strengthening the Reporting of Observational studies in Epidemiology
