# Supplementary material for: Towards inclusive medical education in Egypt: a cross-sectional study of sociocultural adaptation among international students
Source: BMC Med Educ. 2025 Nov 29;25:1672. doi: 10.1186/s12909-025-08232-1 (PMC12676809; doi:10.1186/s12909-025-08232-1)
Supplement: Supplementary file 1 — Supplementary Material 1. [file 12909_2025_8232_MOESM1_ESM.docx]

| **Table 1: Participants’ sociodemographic characteristics (N = 395)** | | | |
| --- | --- | --- | --- |
| **Characteristics** | | | **n (%)** |
| **Overall** |  | | 395 (100) |
| **Age** | 21 or less | | 250 (63.3) |
|  | Older than 21 | | 145 (36.7) |
| **Sex** | Male | | 213 (53.9) |
|  | Female | | 182 (46.1) |
| **Academic year** | 1st year | | 64 (16.2) |
|  | 2nd year | | 98 (24.8) |
|  | 3rd year | | 61 (15.4) |
|  | 4th year | | 89 (22.5) |
|  | 5th year | | 83 (21.0) |
| **Nationality** | Arab country | Syria | 94 (23.8) |
|  |  | Sudan | 70 (17.7) |
|  |  | Palestine | 44 (11.1) |
|  |  | Bahrain | 37 (9.4) |
|  |  | Jordan | 28 (7.1) |
|  |  | Saudi Arabia | 19 (4.8) |
|  |  | Yemen | 15 (3.8) |
|  |  | Algeria | 7 (1.8) |
|  |  | Others* | 13 (3.3) |
|  |  | Total Arabs | 327 (82.8) |
|  | Non-Arab country | Malaysia | 59 (14.9) |
|  |  | Somalia | 3 (0.8) |
|  |  | Nigeria | 3 (0.8) |
|  |  | Philippines | 1 (0.3) |
|  |  | Indonesia | 1 (0.3) |
|  |  | Canada | 1 (0.3) |
|  |  | Total Non-Arabs | 68 (17.2) |
| **Income sufficiency** | Sufficient | | 342 (86.6) |
|  | Less than Sufficient | | 53 (13.4) |
| *Others: Iraq, Kuwait, Lebanon, Libya, South Sudan, and the United Arab Emirates (each had a frequency of 3 or less) | | | |

| **Table 2. Comparison of SCAS-R Scores Across Sociodemographic Characteristics Among International Medical Students at Mansoura University (N = 395)** | | | | | | | |
| --- | --- | --- | --- | --- | --- | --- | --- |
| **Characteristics** | | **Overall sociocultural adaptation^1^** | **Interpersonal Communication^1^** | **Academic/Work Performance^1^** | **Personal Interests & Community Involvement^1^** | **Ecological Adaptation^1^** | **Language Proficiency^1^** |
| **Overall** | | 3.37 (0.69) | 3.38 (0.77) | 3.44 (0.86) | 2.98 (0.88) | 3.28 (0.86) | 4.13 (1.15) |
| **Age** | ≤ 21 | 3.36 (0.65) | 3.37 (0.73) | 3.43 (0.83) | 3.00 (0.85) | 3.27 (0.84) | 4.13 (1.14) |
|  | > 21 | 3.37 (0.76) | 3.40 (0.84) | 3.44 (0.91) | 2.95 (0.92) | 3.29 (0.89) | 4.14 (1.17) |
|  | p-value | 0.931 | 0.706 | 0.934 | 0.584 | 0.851 | 0.925 |
| **Sex** | Male | 3.42 (0.72) | 3.40 (0.80) | 3.49 (0.84) | 3.09 (0.90) | 3.30 (0.89) | 4.25 (1.10) |
|  | Female | 3.31 (0.66) | 3.35 (0.74) | 3.37 (0.87) | 2.86 (0.84) | 3.25 (0.83) | 4.00 (1.19) |
|  | p-value | 0.111 | 0.599 | 0.159 | **0.011** | 0.602 | **0.036** |
| **Academic year** | 1st year | 3.52 (0.72) | 3.51 (0.78) | 3.61 (0.77) | 3.39 (0.92) | 3.50 (0.86) | 3.70 (1.21) |
|  | 2nd year | 3.21 (0.62)**^(a)^** | 3.19 (0.72)**^(b)^** | 3.32 (0.91) | 2.81 (0.74)**^(a)^** | 3.10 (0.75)**^(a)^** | 4.11 (1.15) |
|  | 3rd year | 3.39 (0.50) | 3.35 (0.64) | 3.45 (0.67) | 2.90 (0.69)**^(a)^** | 3.39 (0.69) | 4.34 (0.97)**^(c)^** |
|  | 4th year | 3.31 (0.81) | 3.37 (0.85) | 3.35 (0.93) | 2.96 (0.96)**^(a)^** | 3.14 (1.01) | 4.07 (1.22) |
|  | 5th year | 3.48 (0.71) | 3.52 (0.78) | 3.54 (0.88) | 2.96 (0.96) | 3.38 (0.89) | 4.42 (1.06)**^(c)^** |
|  | p-value | **0.026** | **0.029** | 0.166 | **0.002** | **0.009** | **0.003** |
| **Nationality** | Arab country | 3.40 (0.72) | 3.40 (0.81) | 3.45 (0.91) | 2.97 (0.91) | 3.26 (0.89) | 4.42 (0.97) |
|  | Non-Arab country | 3.21 (0.49) | 3.29 (0.57) | 3.38 (0.56) | 3.03 (0.72) | 3.34 (0.70) | 2.76 (0.97) |
|  | p-value | **0.046** | 0.284 | 0.515 | 0.664 | 0.497 | **<0.001** |
| **Income sufficiency** | Less than Sufficient | 3.40 (0.68) | 3.15 (0.82) | 3.28 (0.95) | 2.80 (0.87) | 2.99 (0.90) | 3.83 (1.24) |
|  | Sufficient | 3.14 (0.73) | 3.41 (0.76) | 3.46 (0.84) | 3.01 (0.88) | 3.32 (0.85) | 4.18 (1.13) |
|  | p-value | **0.011** | **0.021** | 0.161 | 0.097 | **0.008** | **0.039** |
| **^1^**Mean (SD),**^(a)^** significantly less than 1st years, p-value < 0.05, **^(b)^** significantly less than 5th years, p-value < 0.05, **^(c)^** significantly higher than 1st years, p-value < 0.05. | | | | | | | |

| **Table 3. Multivariate Linear Regression of Factors Associated with SCAS-R Scores Among International Medical Students at Mansoura University** | | | | | | |
| --- | --- | --- | --- | --- | --- | --- |
| **Variable** | **Overall sociocultural adaptation^1^** | **Interpersonal Communication^1^** | **Academic/Work Performance^1^** | **Personal Interests & Community Involvement^1^** | **Ecological Adaptation^1^** | **Language Proficiency^1^** |
| **Age (continuous)** | 0.07 (-0.06, 0.19) | 0.07 (-0.06, 0.20) | 0.09 (-0.04, 0.21) | 0.09 (-0.03, 0.22) | 0.04 (-0.08, 0.17) | -0.08 (-0.18, 0.02) |
| **Sex** | -0.07 (-0.17, 0.04) | -0.02 (-0.13, 0.08) | -0.07 (-0.17, 0.03) | **-0.14 (-0.25, -0.04)**** | -0.04 (-0.15, 0.06) | 0.03 (-0.06, 0.11) |
| **Academic year** | -0.04 (-0.17, 0.09) | -0.00 (-0.13, 0.12) | -0.06 (-0.19, 0.06) | **-0.14 (-0.27, -0.02)*** | -0.05 (-0.17, 0.08) | **0.13 (0.03, 0.24)*** |
| **Nationality** | 0.09 (-0.01, 0.20) | 0.05 (-0.05, 0.16) | 0.02 (-0.08, 0.12) | -0.04 (-0.15, 0.06) | -0.03 (-0.14, 0.07) | **0.55 (0.47, 0.64)***** |
| **Income sufficiency** | **0.14 (0.04, 0.24)**** | **0.12 (0.02 , 0.22)*** | 0.08 (-0.02, 0.18) | **0.10 (0.00, 0.20)*** | **0.14 (0.04, 0.24)**** | **0.14 (0.06, 0.22)**** |
| Categorical variables were included in the model as dummy variables: Nationality (0 = Non-Arab, 1 = Arab), Sex (0 = Male, 1 = Female), Academic year (1–5), and Income sufficiency (0 = Less than sufficient, 1 = Sufficient). **^1^** β (95% CI), * p-value<0.05, **p-value<0.01, ***p-value<0.001. CI: Confidence Intervals. | | | | | | |
|  | | | | | | |
